# AI Agents and Agentic AI in plant breeding: new frontiers, opportunities and challenges

**DOI:** 10.3389/fgene.2026.1958764

**Published:** 2026-09-24

**Authors:** Paolo Vitale, Deepak Bhaskar Acharya, Guillermo Gerard, Susanne Dreisigacker, Osval A. Montesinos-López, Abelardo Montesinos-López, Rodomiro Ortiz, José Crossa

**Affiliations:** 1 International Maize and Wheat Improvement Center (CIMMYT), Texcoco, State of Mexico, Mexico; 2 Department of Computer Science, The University of Alabama in Huntsville, Huntsville, AL, United States; 3 Facultad de Telemática, Universidad de Colima, Colima, Mexico; 4 Centro Universitario de Ciencias Exactas e Ingenierías (CUCEI), Universidad de Guadalajara, Guadalajara, Jalisco, Mexico; 5 Department of Plant Breeding, Swedish University of Agricultural Sciences, Alnarp, Sweden; 6 Colegio de Postgraduados, Campus Montecillo, Texcoco, State of Mexico, Mexico

**Keywords:** Agentic AI, AI Agents, Artificial Intelligence (AI), multi-omics integration, plant breeding

## Abstract

Plant breeding increasingly integrates diverse data sources, including genomic, phenomic, and environmental information, yet current artificial intelligence (AI) applications remain largely isolated analytical tools. Coordinating the entire breeding pipeline, from data collection and quality control to analysis, prediction, and decision-making, remains a challenge. Here we hypothesize that AI Agents and Agentic AI systems, which autonomously execute tasks using reasoning, memory, and tool integration, may orchestrate complex breeding objectives by coordinating specialized agents across multiple data domains. This approach has the potential to enhance breeding efficiency, reduce operational errors, and accelerate genetic gain by connecting previously fragmented processes. Although empirical validations remain forthcoming, the framework presented here provides a conceptual foundation for future implementation and evaluation. While technical, ethical, and institutional challenges exist, implementing such AI frameworks could support breeders in managing the complexity of modern genetic improvement programs, ultimately contributing to enhanced cultivar development and food security.

## Introduction

Plant breeding is the process of genetically improving plants to benefit humanity (Bernardo, 2020). This long-term, multi-cycle endeavor combines biological knowledge, field experimentation, quantitative genetics, prediction models, and breeders’ expertise to increase genetic gain and deliver superior cultivars. Plant breeding has played a pivotal role in ensuring global food security by increasing crop productivity to meet the growing food demand driven by population growth and by developing crop varieties better adapted to the challenges posed by climate change (Qaim, 2020; Xiong et al., 2022). Over the past 50 years, breeding strategies, enabling tools and data accessibility have evolved dramatically, shifting from crossbreeding and selection methods to advanced protocols relying on knowledge from cell and molecular biology as well as tools for high-throughput phenotyping (HTP) and data analysis (Ahmar et al., 2020). The application of quantitative genetics principles such as randomization, robust experimental designs, analyses across single- and multi-environment trial (MET), selection indices (strategies that combine multiple trait values into a single score for selection purpose), and optimal contribution selection (OCS), which balances genetic gains with the maintenance of genetic diversity, has enhanced the precision of selection and the overall success of breeding programs. The use of genotyping technology that support approaches such as marker-assisted selection (MAS) have improved the accuracy of selection for qualitative inherited traits (Ribaut and Hoisington, 1998). More recently, genomic selection (GS) has become central to plant breeding, using genome-wide molecular markers in statistical models to predict complex quantitative traits by capturing both major- and minor-effect quantitative trait loci (QTL) (Meuwissen et al., 2001). This enables breeders to select untested lines based on predicted trait values or genomic estimated breeding values before field testing, thereby accelerating the breeding cycle (Desta and Ortiz, 2014). GS has been successfully applied across a wide range of crops such as maize, wheat, sorghum, rice, and potato, for traits including yield, quality, biomass, and host plant resistance to pathogens (Crossa et al., 2013; Battenfield et al., 2016; Zhang et al., 2017; Enciso-Rodriguez et al., 2018; Fernandes et al., 2018; Spindel and Iwata, 2018; Stich and Van Inghelandt, 2018; Huang et al., 2019; Rice and Lipka, 2019; Dreisigacker et al., 2023; Vitale et al., 2025). Historical pedigree data have also proven valuable for predicting target lines, sometimes matching the accuracy of genomics-based predictions (Crossa et al., 2010). Similarly, phenomic selection (PS) uses HTP data instead of molecular markers to predict candidate genotypes (Rincent et al., 2018). Enviromics, which incorporates environmental parameters, helps estimate similarities within target environments and better understand genotype-by-environment ( G×E ) interactions, thereby improving the accuracy of selecting new candidate lines (Resende et al., 2021).

Modern plant breeding is characterized by the need to coordinate various interconnected decisions rather than relying on a single data source or prediction model. Breeding programs must determine which crosses to make, which individuals within segregating families to advance, which lines to phenotype, and which environments to sample. Additional considerations include selecting appropriate field designs, correcting field heterogeneity, analyzing multi-environment trials, integrating pedigree and genomic information, implementing sparse testing, incorporating phenomic and environmental covariates, and efficiently recycling selected parents into subsequent cycles. These decisions are highly interdependent: for example, a poor field design can compromise downstream genomic prediction, mislabeled seed or tissue samples can reduce genetic gain, inadequate spatial adjustment can distort phenotypic values, and poorly planned sparse testing can diminish the value of multi-environment information. This complexity underscores the need for integrated, coordinated solutions in modern plant breeding.

Recently, it has become clear that integrating genomics, phenomics, and enviromics data is an effective strategy for increasing selection accuracy and the adaptability of genotypes, thereby accelerating genetic gain in plant breeding (Crossa et al., 2021; Li et al., 2026). Building on this, multi-omics approaches that also incorporate transcriptomics, metabolomics, proteomics, and epigenomics are being explore for their potential to further enhance genetic gain (Mahmood et al., 2022).

Although conventional statistical models like genomic best linear unbiased prediction (GBLUP) have been successful in genomic selection (GS), they struggle to manage and integrate the increasingly complex relationships among diverse omics data and phenotypic outcomes (Xue et al., 2026). Therefore, more complex algorithms are naturally required. In this context, AI-based models have a role to play (Crossa et al., 2025b). AI models, such as machine learning (ML) and deep learning (DL), can handle more efficiently the complex interactions among multiple omics and plant phenotypes to deliver more accurate predictions (Montesinos-López et al., 2021; Montesinos-López et al., 2024a). However, most current applications of AI in plant breeding are still model-centered, with each model typically trained for a specific analytical task such as yield prediction, disease classification, genome-wide association analysis, genomic selection, image-based phenotyping, or cross-recommendation.

AI agents and Agentic AI represent a different conceptual shift from traditional AI models. While an AI model is designed to make predictions, an AI agent can perform tasks by utilizing tools, memory, reasoning, and external information. An agentic AI system goes further by coordinating multiple specialized agents to achieve broader goals through planning, task decomposition, monitoring, and adaptation (Yao et al., 2023; Masterman et al., 2024). This distinction is particularly important in plant breeding, where the process involves a coordinated sequence of field, laboratory, statistical, logistical, and strategic decisions spanning multiple years, locations, generations, and product targets, rather than a single prediction problem.

While AI agents and Agentic AI systems have been applied in fields such as biomedicine, healthcare, chemistry, finance, education, logistics, and urban digital twins, plant breeding presents a unique challenge. Unlike other domains where the decision unit may be a patient, molecule, transaction, document, or route, plant breeding involves managing a complex pipeline across populations, environments, generations, and selection cycles. Although examples from other fields are useful because they show that AI agents can coordinate specialized tools, retrieve external information, integrate heterogeneous data, and support complex decisions, the key question for this perspective is not whether Agentic AI can function in other domains, but rather how it may be practically implemented in modern plant breeding. Indeed, it is important to note that while the examples of AI agents and Agentic AI discussed in this perspective come from empirical applications in other fields, their implementation in plant breeding remains hypothetical. The framework proposed here represents a conceptual roadmap rather than an experimentally validated system.

Therefore, this perspective article has four objectives: (1) to provide an overview of the current state of the art of the use of AI in plant breeding; (2) to distinguish conventional AI models, AI agents, and Agentic AI systems; (3) to hypothesize an operational framework for implementing AI Agents and Agentic AI in modern breeding pipelines across various breeding tasks: (4) to discuss the technical, biological, institutional, and ethical challenges that must be addressed before AI Agent and Agentic AI can be responsibly deployed in breeding programs.

## AI-driven plant breeding–State of the art overview

In recent years, a copious amount of research has been produced on the application of AI in plant breeding (Yoosefzadeh Najafabadi et al., 2023; Cheng and Wang, 2024; Eftekhari et al., 2024; Fang, 2024; Farooq et al., 2024; Crossa et al., 2025a; Debnath et al., 2025; Fu et al., 2025; Sangjan et al., 2025; Tanin et al., 2026). AI-based models can leverage data from a single omics layer or multiple layers to discern omics interactions. AI-based models, particularly multimodal DL, have been used to jointly leverage genomic and phenomic information to improve grain yield predictability, potentially increasing selection accuracy in wheat breeding programs (Togninalli et al., 2023). It has been observed that DL approaches consistently outperform conventional Bayesian statistics for predicting phenology-related traits in both single- and multi-trait strategies in maize breeding (Mora-Poblete et al., 2023). Montesinos-López et al. (2024b) conducted a multi-omics study of winter wheat using genomics, phenomics, and enviromics. Interestingly, the authors observed a ∼50% improvement in predictability when enviromics was included and concluded their research with empirical evidence that incorporating additional information into the AI-based model can increase predictability compared with using a single omics approach. Additionally, OpenPheno has been developed as a smartphone-based application that includes the WheatHeadPheno tool among others (Hu et al., 2025). This last uses RGB (red, green, and blue) images collected with a conventional smartphone and a DL framework to predict several yield-related traits. For wheat spike count, promising predictive results were achieved (*R* ^2^ = 0.65). Unmanned Aerial Vehicle (UAV)-derived data have been used to predict rice yield using several DL models (Zhou et al., 2025). Interestingly, the authors observed promising predictive performance (*R* ^2^ = 0.73) when a 2-dimensional convolutional neural network (CNN) was applied. It has been shown that DL can leverage structural genomic variants and single-nucleotide polymorphisms (SNPs) to predict yield in rice, outperforming conventional Bayesian models in 75% of study cases (Vourlaki et al., 2024). A new CNN-based DL model (SoyDNGP) has been tested for several traits in soybean and other species, including cotton, maize, rice, and tomato (Gao et al., 2023). It has been found that SoyDNGP consistently outperforms other AI-based models, such as DNNGP and DeepGS, in trait predictability. It has been demonstrated that AI, such as You Look Only Once (YOLO) DL models, can predict fruit and foliar diseases in crops, including peach and strawberry, using image input (Alhwaiti et al., 2025). Interestingly, YOLO version four achieved 98% accuracy in detecting the correct diseases. In the context of plant disease, it has been discussed how AI-based algorithms can harmonize multi-omics data, such as genomics and phenomics, to predict plant disease-resistant phenotypes, facilitating the transition from old-fashioned to AI-driven breeding (Ma et al., 2025). Indeed, yellow rust severity has been successfully predicted in bread and durum wheat by jointly using genomics and HTP-derived data through AI-based frameworks (Vitale et al., 2026). AI approaches have also recently been used to optimize cross-scheme in wheat (Michel et al., 2025). Interestingly, this integrated AI tool, trained by the previous breeder’s decisions, can significantly reduce the time and effort required to identify the best-performing crosses. The use of generative AI (genAI) in breeding has recently been discussed (Pérez-Enciso et al., 2025). It has been suggested that genAI can be used alongside stochastic simulations, where conventional methods simulate novel genotypes and genAI produces synthetic data for genotypes and environments.

While AI-based models can be the main engine for delivering accurate predictions, they can also serve as an orchestrator, as with Large Language Models (LLMs). Indeed, the BOLE AI Agent framework has recently been shown to perform genomics-based tasks, such as genome-wide association studies (GWAS) and GS (Wang et al., 2026) in plant breeding. While no trace of Agentic AI systems operating in plant breeding is present; to date, this is the most recent use of an AI agent in plant breeding. This successfully integrated LLMs with conventional statistical models to enhance the breeding analytics pipeline. However, to date, no Agentic AI applications have been performed or discussed in plant breeding yet. Table 1 reports the paragraph summary of the some of the most recent applications of ML and DL in plant breeding.

**TABLE 1:** Examples of recent machine- and deep-learning models in plant breeding.

Crop	Tool/Model	Data type	Task/Traits	Key finding	References
Wheat	PheGeMIL (multimodal DL)*	Genomics (SNPs) + phenomics (UAV multispectral, thermal, DEM)	Grain yield prediction	PCC = 0.754 ± 0.024; outperforms lasso, ridge, RF, GBM	Togninalli et al., 2023
Maize	Deep learning (multi-trait, multi-environment)	Genomics (SNPs)	Flowering traits (ASI, FF, MF)	+14.4% accuracy (multi-trait vs. single-trait); outperforms bayesian models	Mora-Poblete et al., 2023
Winter wheat	Multi-omics framework (GBLUP + DL)	Genomics + phenomics + enviromics	Multi-omics genomic prediction	∼50% improvement in predictability with enviromics included	Montesinos-López et al., 2024b
Wheat	WheatHeadPheno/OpenPheno (DL, RGB images)	Phenomics (RGB smartphone images)	Yield-related traits; spike count	*R* ^2^ = 0.65 for spike count	Hu et al., 2025
Rice	2D CNN (UAV-derived data)	Phenomics (UAV imagery)	Yield prediction	*R* ^2^ = 0.73	Zhou et al., 2025
Rice	MLP + CNN (DL)	Genomics (SNPs + structural variants: SVs, TIPs, DEL, DUP, INV)	Multi-trait genomic prediction (yield, binary traits)	DL outperforms bayesian in 75% of cases; SVs improve prediction over SNPs alone	Vourlaki et al., 2024
Soybean, cotton, maize, rice, tomato	SoyDNGP (CNN-based)	Genomics (SNPs)	Multi-trait genomic prediction	Outperforms DNNGP and DeepGS across species	Gao et al., 2023
Peach, strawberry	YOLO v4 (DL, image-based)	Phenomics (RGB images)	Fruit and foliar disease detection	98% disease detection accuracy	Alhwaiti et al., 2025
Multiple crops	AI framework (genomics + phenomics)	Genomics + phenomics	Disease-resistant phenotype prediction	Framework for AI-driven disease resistance breeding	Ma et al., 2025
Bread and durum wheat	AI framework (genomics + HTP)	Genomics + phenomics (HTP)	Yellow rust severity prediction	Successful joint prediction using genomic + HTP data	Vitale et al., 2026
Wheat	AI cross-scheme optimizer (trained on breeder decisions)	Historical selection data + genomics	Cross selection optimization	Significant reduction in time and effort for identifying best crosses	Michel et al., 2025
Multiple crops	Generative AI + stochastic simulation	Genomics + enviromics (synthetic)	Synthetic genotype and environment data generation	GenAI can augment simulation pipelines with synthetic data	Pérez-Enciso et al., 2025
Plant breeding (general)	BOLE AI agent (LLM + statistical models)	Genomics	GWAS and genomic selection	First AI agent in plant breeding; integrates LLMs with conventional GS models	Wang et al., 2026

* ASI: Anthesis-Silking Interval; CNN: convolutional neural network; DEL: deletions; DEM: digital elevation model; DL: deep learning; DNNGP: deep neural network genomic prediction; DUP: duplications; FF: female flowering; GBM: gradient boosting machine; GenAI: generative artificial intelligence; GBLUP: genomic best linear unbiased prediction; GS: genomic selection; GWAS: Genome-Wide Association Study; HTP: High-Throughput Phenotyping; INV: inversions; LLM: large language model; MF: male flowering; MLP: multilayer perceptron; PCC: pearson correlation coefficient; PheGeMIL: Phenotype–Genotype Mutual Information Learning; RF: random forest; RGB: red, Green, Blue; SNPs: Single Nucleotide Polymorphisms; SVs: Structural Variants; TIPs: Transposable Element Insertion Polymorphisms; UAV: unmanned aerial vehicle; YOLO: you only look once.

## AI models, AI agent, and agentic AI: principles and differences

AI is rapidly reshaping several sectors, including healthcare, architecture, manufacturing, education, transport, agriculture, and finance (Sijing and Lan, 2018; Clara Eli-Chukwu, 2019; Arinez et al., 2020; Shaheen, 2021; Cao, 2023; Ploennigs and Berger, 2023; Dhaliwal, 2024). The increasing use of AI in plant breeding requires a clear distinction among three related but different concepts: AI models, AI agents, and Agentic AI systems. These terms are sometimes used interchangeably, but they imply different levels of autonomy, responsibility, integration, and decision support. An AI model is usually designed to perform a specific analytical task. These were mostly described in the previous section. These tasks may include predicting grain yield from genomic markers, classifying disease symptoms from images, estimating flowering time from environmental covariates, extracting phenomic features from UAV images, predicting genomic estimated breeding values, or integrating omics data for trait prediction.

While AI Agents and Agentic AIs are based on AI algorithms; the literature provides a clear distinction between both (Sapkota et al., 2026). AI Agents are typically oriented toward specific objectives or tasks and operate within a confined environment (Sado et al., 2023). These systems evolved from operating in confined, rule-based environments to learning-driven, flexible, and adaptive architectures in the post-LLM era (Hughes et al., 2025). These agents enhanced LLMs with capabilities for using external tools function calling, and sequential reasoning, enabling them to retrieve real-time information and execute multi-step workflows autonomously (Liu et al., 2024). Interestingly, each agent plays a role and has access to a variety of tools that help them accomplish their job, either independently or as part of a team. Some agents also contain a memory component that allows them to save and load information outside their messages and prompts (Masterman et al., 2024). AI agents need strong reasoning capabilities to effectively interact with complex environments, make autonomous decisions, and assist humans across a wide range of tasks. This integration between “acting” and “reasoning” allows new tasks to be learned quickly and enables robust reasoning, even under previously unfamiliar circumstances or information uncertainties (Yao et al., 2023). However, AI agents showed several limitations that can affect their scalability for complex and dynamic tasks such as difficulties in maintaining coherent long-term task planning, unreliable tool selection in multi-step workflows, and the lack of flexibility (Feriani and Hossain, 2021; Feng et al., 2025).

Agentic AIs represent a continuum of AI Agents. These are goal-oriented algorithms that exhibit high flexibility, enabling them to operate in dynamic environments, including real-world scenarios, and to make autonomous decisions (Acharya et al., 2025). These systems represent a new category of intelligent architecture in which multiple specialized agents collaborate to achieve complex and dynamic objectives, utilizing collaborative reasoning and multi-step planning (Sapkota et al., 2026). Agentic AIs are characterized by continuous learning driven by Reinforcement Learning (RL), which enables these systems to interact productively with the environment and adapt their strategies to achieve goals over time (Matsuo et al., 2022). Indeed, the adaptive control mechanism allows the agent AI to respond to external changes and to adapt consequently (Zhao et al., 2021). Unlike AI agents built for specific tasks, Agentic AI’s goal-oriented architecture enables agents to prioritize and pursue multiple objectives simultaneously (Binucci et al., 2022). This allows these systems to manage complex objectives (Acharya et al., 2025). Indeed, when users launch a complex objective, these systems can autonomously split it into simpler, smaller ones through goal decomposition (Bornet and Wirtz, 2025). These smaller tasks are organized by the multi-step reasoning and planning mechanisms that characterize the Agentic AI system.

In summary, while AI Agents are built to perform a single specific task, Agentic AI systems can manage complex objectives by decomposing them into smaller tasks (Figure 1). AI Agents offer little autonomy and require high levels of supervision, whereas Agentic AIs are characterized by high autonomy and low levels of human intervention. AI Agents usually work independently, whereas Agentic AIs orchestrate multi-agent systems collaboratively. AI Agents showed a limited capability to learn and within their restricted environment, whereas Agentic AI can adapt to changing environments and reset strategies.

**FIGURE 1:**
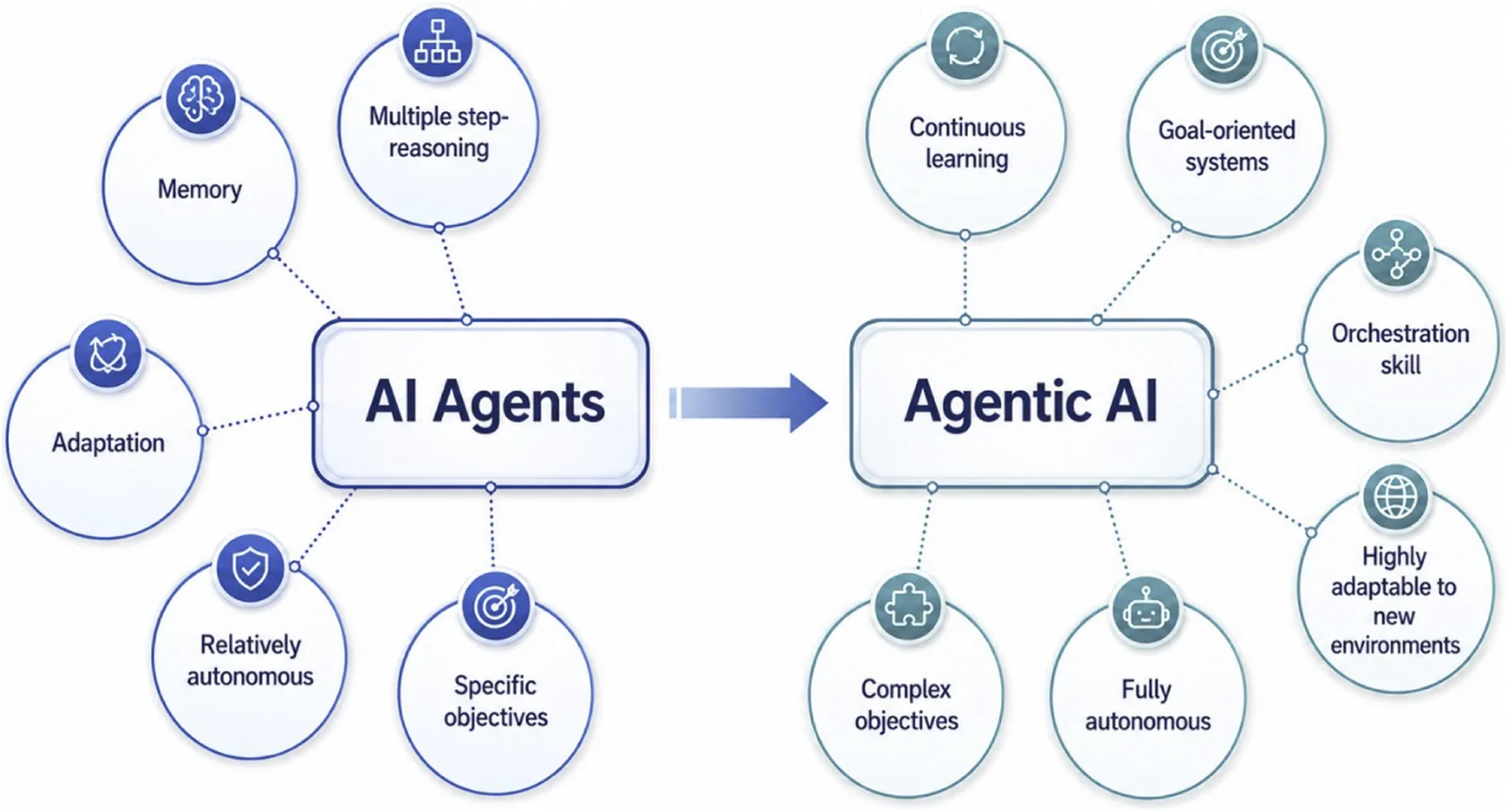
Comparison of AI Agents and Agentic AI.

### Case studies

While in plant breeding, the use of AI Agents and Agentic AI applications is rare or nonexistent, these systems are widely used in other fields. For instance, a new AI agent-based intelligent urban digital twin has been developed to integrate, analyze, and predict energy consumption in urban contexts (Choi and Yoon, 2025). Existing applications of single AI agents typically fall into four categories: automated handling of structured customer support and enterprise search queries; classification-based filtering and prioritization of high-volume email traffic; behavior-driven personalization and basic automated data reporting; and scheduling assistants capable of interpreting calendars and booking tasks with limited user input (Sapkota et al., 2026). In biomedicine, an Agentic AI called Biomni has been developed to perform several complex tasks, including causal gene prioritization, drug repurposing, rare disease diagnosis, microbiome analysis, and molecular cloning, and to automate and enhance biomedical research across a wide range of subfields (Huang et al., 2025). Interestingly, the system can autonomously formulate novel, testable hypotheses, perform complex bioinformatics analyses, and design new experimental protocols. Powered by an LLM, it can mine essential tools, databases, and protocols from tens of thousands of publications across 25 biomedical domains. Agentic AI can operate in the field of energy by optimizing consumption and predicting demand, in the field of transport by improving route planning and reducing delivery risks, in the field of healthcare by boosting medical diagnosis and improving patient data management, and in the field of finance by analyzing market trends and optimizing investment decision-making (Hosseini and Seilani, 2025). It has been discussed that Agentic AI has the potential to redefine healthcare across various aspects of the field, including diagnostics, clinical decision support, treatment planning, patient monitoring, administrative operations, drug discovery, and robotic-assisted surgery (Karunanayake, 2025). Agentic AIs have also been used to target educational content, serving as a tool for educators and researchers to integrate AI into education (Heilala et al., 2023).

## Perspectives in plant breeding

### Experimental design and field allocation resources

The first specialized component could be an experimental design and field allocation agent. This agent would recommend field designs based on the breeding stage, number of entries, seed availability, field size, number of locations, number of checks, expected spatial heterogeneity, and the intended downstream analysis. In early generations with thousands of lines and limited seed, the agent might recommend augmented designs, partially replicated designs, incomplete-block designs, or sparse-testing layouts. In later-stage trials, it might recommend alpha-lattice or multi-location replicated trials (Cullis et al., 2006; Piepho et al., 2008). The agent would not simply choose a design; it would explain why the design is appropriate for the breeding objective and what consequences it has for prediction accuracy, field precision, and resource allocation.

Experimental design has been extensively explored to optimize phenotypic data collection in plant breeding (Fisher and Fisher, 1966; Qiao et al., 2000; Singh and Bhatia, 2017; dos Santos et al., 2024). However, as breeding programs increasingly incorporate novel data layers such as microbiome, metabolome, or other omics, the question arises whether designs optimized for agronomic traits remain appropriate for these fundamentally different data types. Sampling requirements, spatial dependencies, and cost structures differ substantially across omics layers, making a one-size-fits-all design approach suboptimal. AI agents could support breeders in navigating this complexity by recommending the most appropriate experimental design based on the trial objective, the data type to be collected, and the available resources.

Such an agent could also coordinate sparse testing, one of the most important applications for modern breeding. Sparse testing allows breeding programs to phenotype only a subset of G×E combinations while borrowing information through pedigree, genomic relationships, environmental similarity, and multi-environment models (Jarquin et al., 2020). In early generations, this is especially valuable because breeders may have many segregating lines but limited seed, field space, and budget. An experimental-design agent could decide which lines should be tested in which environments, which checks should be included, and how connectivity should be maintained across trials. This would allow breeders to evaluate more material with fewer plots per line and to discard inferior material earlier.

### Data quality and traceability

A second component would be a field data quality and traceability agent. In plant breeding programs, large amounts of data are collected every year. Traditional methods for collecting data are error-prone, and without a centralized system, can result in poor data management and the loss of crucial data. In recent years, systems such as FieldBook and PhenoApp have been developed to improve data collection in plant breeding (Rife and Poland, 2014; Röckel et al., 2022). Additionally, systems such as AGROBASE/4 and the Enterprise Breeding System (EBS) (https://excellenceinbreeding.org/toolbox/tools/enterprise-breeding-system-ebs) have been developed to improve data management in plant breeding (Mulitze, 1990). However, current data management systems in breeding (FieldBook, EBS, *etc.*) are built to collect and store data, not to validate it. Nevertheless, these are limited to phenotypic records, not to multi-omics data generated within a breeding program. Errors can occur during phenotypic recording due to operator error. Similarly, mistakes can occur at the genomic level, e.g., during tissue collection, DNA extraction, and genotyping. A simulation study has shown that mislabeling can occur in plant breeding and can affect GS prediction accuracy and, consequently, genetic gain (Yabe et al., 2018). As for genomics, this can be extended to other omics, including metabolomics, transcriptomics, and proteomics. Additionally, phenomics-based data, such as images captured by a UAV or a smartphone, can be mislabeled or collected without adhering to standard operating procedures (SOPs). Therefore, an AI agent that can oversee data quality control across multiple omics and select high-quality information from the data lake for processing and subsequent decision-making would likely benefit the overall breeding pipeline. While such an application does not yet exist in plant breeding, AI agents for data quality control oversight have been demonstrated in other data-intensive fields. In clinical research, multi-agent frameworks have been developed specifically for error detection and correction in medical records (Corbeil, 2024). Similarly, human error, inconsistent formats, or a lack of standardization can lead to inaccurate recommendations and flawed analyses in healthcare. It has been discussed how the continuous monitoring by AI Agents of data inputs for discrepancies and errors enables healthcare organizations to intervene promptly before inaccuracies propagate through the system. Beyond improving the reliability of clinical insights, such quality assurance mechanisms have demonstrated tangible benefits for both patient outcomes and operational efficiency (Pottla, 2025).

### Workflow optimization

Several types of analysis are usually performed within a breeding program. From the experimental design to trial analysis, from GS to parental selection and cross-prediction, and from HTP-derived data to pedigree-based selection. Many of these analyses use specific software or packages, often without communicating with one another. Collectively, these aim to improve predictability and selection accuracy, and ultimately to maximize genetic gain. However, it is important to distinguish between these concepts, as they are related but not equivalent outcomes. Predictability refers to the correlation between predicted and observed phenotypic values and can be empirically estimated from data. Prediction accuracy refers to the correlation between predicted and true breeding values, which are unknown by definition. In a GS-based breeding program, prediction accuracy corresponds to selection accuracy, which directly determines the rate of genetic gain: the higher the selection accuracy, the greater the expected genetic gain achievable per breeding cycle.

Field trials are affected by soil heterogeneity, water gradients, fertility differences, border effects, disease patches, and other spatial sources of variation. If these effects are not corrected, phenotypic values used for genomic prediction may reflect field noise rather than genetic signal. A spatial analysis agent could automatically evaluate each trial, inspect residual patterns, compare alternative spatial mixed models, identify outliers, estimate adjusted means, and generate best linear unbiased estimates and predictors (BLUEs and BLUPs) for downstream analyses (Rodríguez-Álvarez et al., 2018).

Similarly, modern breeding programs test material across locations, years, management systems, and stress conditions. The goal is not only to estimate mean performance, but also to understand G×E interaction, stability, adaptation, and the target population of environments (Malosetti et al., 2013; Jarquín et al., 2014). This agent could integrate trial data with weather, soil, management, disease pressure, and crop-growth descriptors. It could estimate environmental similarity, identify environmental clusters, summarize stress periods, and define biologically meaningful windows for environmental covariates.

Subsequently, a GS agent could integrate pedigree, genomic markers, sparse phenotypes, environmental covariates, and historical trial data. It could compare models and strategies before the implementation, such as pedigree-based BLUP, GBLUP, Bayesian models, genomic-environment interaction models, kernel methods, and multimodal models. It could evaluate whether predictions are reliable for within-family, across-environment, or forward predictions into the next breeding cycle. Most importantly, it could update the training set as new data arrives over time. Specialized analyses require skilled professionals and cannot be run by breeders alone. User-friendly software such as CHiDO and GS4PB has recently been developed to facilitate complex analyses, such as GS, in single- and multi-environmental trials and single- and multi-trait within a plant breeding program (González et al., 2024; Ramasubramanian et al., 2025). Despite the utility of such software, it remains hard to build user-friendly software that encompasses all the steps and manages multiple data sources in plant breeding. Having an AI agent to manage a single analysis, such as trial analysis, GS, or multi-omics selection, could enhance the optimization and facilitate the data analysis workflow in breeding. A first example has recently shown how an AI agent can simplify GS and GWAS in a breeding program (Wang et al., 2026). These kinds of Agents can make analysis easier and faster while delivering actionable results for non-expert users.

In a more general framework, PLLaMa is an open-source large language model specifically tailored to plant science by leveraging domain-specific literature and knowledge. The model is designed to provide more accurate and context-aware responses than general-purpose LLMs for plant biology and breeding applications. It supports literature synthesis, knowledge retrieval, hypothesis generation, and scientific reasoning. By offering a transparent and customizable framework, PLLaMa seeks to accelerate research and promote the adoption of AI-driven tools in plant science (Yang et al., 2024).

Interestingly, in other fields such as chemistry, an AI Agent has been developed to accomplish tasks across organic synthesis, drug discovery, and materials design (Bran et al., 2023). By integrating 18 expert design tools, the agent can autonomously plan and execute the synthesis of an insect repellent and three organocatalysts, and guide the discovery of a novel chromophore, demonstrating enormous potential to optimize the task workflow in chemistry. In biology, a multi-AI agent called CellAgent can process and execute single-cell RNA sequencing data analysis tasks (Xiao et al., 2024). This is capable of automating the framework, dramatically reducing the workload for scientific data analysis, and entering the “Agent for Science” era.

### Omics integration

Multi-omics represents the near future of plant breeding. Plant breeders deal with complex traits that are rarely explained by a single omics at a time. Therefore, unimodal prediction models often fail to explain all the trait variance. For this reason, multimodal models that can embrace multi-omics data, including genomic markers, environmental variables, HTP-derived data, pedigree information, and physiological variables, often outperform unimodal models in predicting complex traits (Crossa et al., 2026). The superiority of DL models in managing multiple omics has been assessed in plant breeding (Cembrowska-Lech et al., 2023; Wang et al., 2023; Gao et al., 2025). However, the presence of AI Agents or Agentic AIs capable of orchestrating multimodal analysis across different omics layers is lacking. These tools could perform model selection through validation, identify the omics most meaningful for a target trait, and deliver actionable results to breeders, thereby achieving higher selection accuracy. In medicine, it has been discussed that Agentic AI tools can integrate genomics and transcriptomics data, representing a shift from passive text generation to autonomous analytical orchestration (Rozen et al., 2026). Still in the field of medicine, an AI Agent called AutoBA (Automatic Bioinformatics Analysis) has been developed for a fully automated multi-omics analysis. AutoBA can implement the analytical bioinformatics process with minimal user input, delivering detailed, step-by-step plans for various tasks (Zhou et al., 2024). Similarly, another AI Agent, BiOmics, has been developed to address multiple omics in medicine (Cao et al., 2026). BiOmics successfully covers indirect pathogenic variants, achieves reference-free cell annotation, and prioritizes drug-repurposing candidates tailored to specific datasets, showing a paradigm shift from descriptive “data fitting” to autonomous, knowledge-driven interpretation. The use of AI-based tools for integrating omics data is also reshaping the field of medicine. AI Agents and Agentic AIs can be used to orchestrate multi-omics analyses and automatically and autonomously retrieve, analyze, and synthesize omics data, literature, and prior knowledge to generate testable hypotheses and design experiments to validate them (Li et al., 2026). The agent can use foundation models to perform omics analyses, uncover key disease targets and pathways, and predict perturbation targets and drugs for disease prevention and treatment.

### Parent selection and crossing design

Once candidate lines are evaluated, breeders must decide which lines to advance, which to use as parents, and which crosses to make. This is a multi-objective problem. While parental and cross-prediction methods already exist, an integrating strategy that considers multiple data sources is missing (Mohammadi et al., 2015; Gorjanc and Hickey, 2018; Villar-Hernández et al., 2018). Short-term gain must be balanced against genetic diversity, co-ancestry, disease resistance, quality, adaptation, market requirements, and long-term genetic gain. A parent-selection agent could combine GEBVs, pedigree, genomic relationships, historical selection data, heterotic patterns, environmental characteristics of the target areas, trait correlations, selection indices, optimal contribution selection, and target product profile constraints. It could propose crossing plans that maximize expected gain while controlling inbreeding and preserving useful diversity. This component is especially important because Agentic AI systems may otherwise optimize too aggressively for short-term targets. A crossing design agent must include explicit constraints on diversity, inbreeding, effective population size, trait trade-offs, and uncertainty.

### Enhancing decision-making

To date, breeding programs that leverage GS, HTP-derived data, environmental parameters, and other information typically use these outcomes in isolation. In the context of line advancement, breeders often separately examine the performance of candidate lines for several traits, the pedigree data, and eventual vegetative indices produced by HTP. Then, based on the target product profile TPP, they elaborate on all the information and perform the selection. Additionally, breeders can overlook pedigree data, predicted values for unknown lines, and field performance of observed lines when choosing crosses. However, it is nearly impossible for any human brain to digest all the data sources of information and make clear decisions. Based on an ideotype or TPP, Agentic AIs could coordinate different AI Agents, integrating all available layers of information to pursue these objectives and enhance the breeding selection process. These could orchestrate different AI Agents working on separate data, molecular marker information, phenomics data, pedigree information, environmental covariates, any eventual omics layer, and the history of selection since the beginning of the program and then merge the results to boost selection (Figure 2).

**FIGURE 2:**
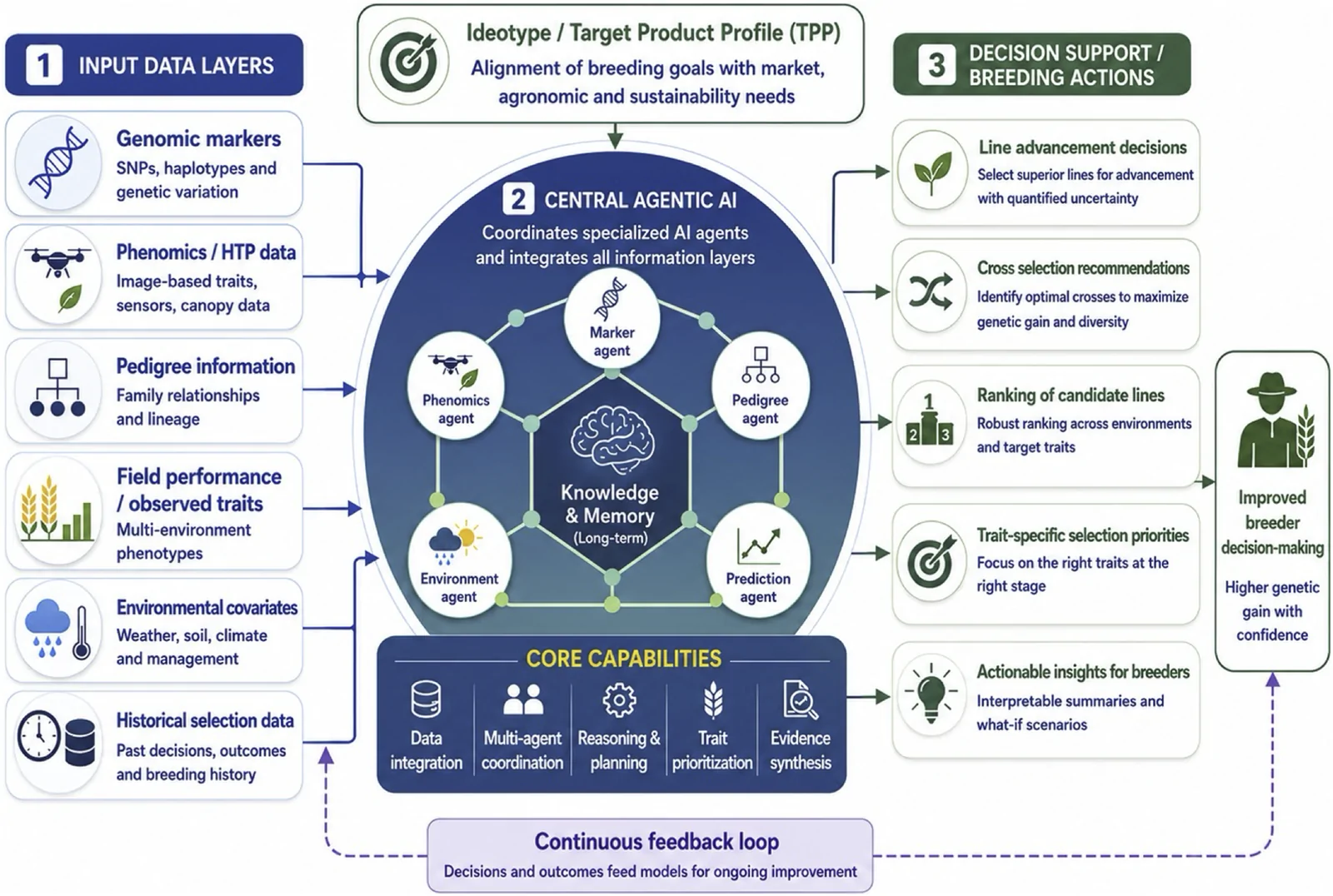
The central role of an Agentic-AI system in improving plant-breeding efficiency.

The ability of Agentic AIs to enhance the decision-making process has already been discussed in the literature (Hughes et al., 2025). Agentic AI can enhance decision-making by integrating heterogeneous data sources, as demonstrated in other fields. In oncology, an autonomous AI agent has been developed and validated, capable of supporting personalized clinical decision-making by coordinating specialized tools for histopathology image analysis, radiological image segmentation, genomic data processing, and retrieval of medical guidelines (Ferber et al., 2025). This reached correct clinical conclusions in 91% of cases and improved decision-making accuracy from 30.3% to 87.2% compared to a standalone language model. In supply chain management, Agentic AI systems have been shown to autonomously coordinate consensus-seeking decisions among multiple actors operating in dynamic, uncertain data environments, thereby reducing the need for human intervention in complex, multi-step decision processes (Jannelli et al., 2025). In healthcare, it has been discussed how Agentic AIs can support medical diagnosis by analyzing historical data, proactively automating administrative tasks, describing prescriptions to patients, and providing 24/7 support (Pati, 2025). It has also been widely shown that Agentic AI can enhance decision-making in finance. These systems can analyze real-time data and make autonomous investment decisions, thereby revolutionizing investment strategies (Dixon and Halperin, 2020; Rong Wang et al., 2023).

### Operational optimization

Beyond data analysis and management, as addressed in the previous sections, agentic AI is increasingly integrated with robotics and remote sensing to automate physical tasks throughout the breeding pipeline. The robotic functions in agriculture have already been discussed (Duckett et al., 2018). Interestingly, AI and robotics can be integrated to support planting, plant care, harvesting, and post-harvesting (Gupta and Gupta, 2024). Additionally, robotics, remote sensing, and AI are drastically changing the concept of trait collection in plant breeding (Fu et al., 2025). A ground-based robot can collect trait data at close range, while UAVs can collect trait data rapidly and at a large scale (Xu and Li, 2022; Gano et al., 2023). Similarly, high-resolution satellite imagery can be used to collect crop traits, thereby increasing throughput, efficiency, and objectivity during field phenotyping (Zhang et al., 2020). In the field of molecular biology, robotic and AI tools represent a significant advancement in the automated extraction of nucleic acids and library preparation, addressing complexity and diversity while minimizing human involvement (Lu et al., 2025). This autonomously produces process flowcharts, predetermined reagents and consumable quantities, and aligns process steps with specific module actions via strategy-guided segmented program file arrangements.

### Non-technical domains

Plant breeding presents a complex architecture; beyond all the technical domains, several domains are necessary to carry out a breeding program, including administration, project management, finance, and market intelligence. Across non-technical domains, AI agents are already demonstrating concrete capabilities. In administration, LLM-based agents have been benchmarked on realistic office workflows spanning document processing, email management, calendar scheduling, and cross-application task execution, demonstrating the potential to automate complex multi-step administrative processes (Wang et al., 2024). In project management, AI agents have demonstrated the ability to autonomously monitor task progress, detect bottlenecks, reallocate resources, and forecast risks across complex multi-team workflows, and evidence confirming improvements in cost estimation, duration forecasting, and risk assessment across multiple project phases (Adamantiadou and Tsironis, 2025). The role of AI Agents in market intelligence has been widely discussed, highlighting the potential of these systems to improve customer engagement and increase the impact of marketing campaigns, while balancing economic goals with the need for environmental sustainability and social responsibility (Emon and Khan, 2024).

## Impact of AI on the breeding job market

AI Agents and Agentic AI are beginning to reshape how work is organized across these domains, not only in data analysis but also in human resource management, decision-making, and the execution of physical tasks (Vrontis et al., 2022). The use of AI in collaborative working environments has become vital across diverse sectors, enhancing efficiency, creativity, and innovation (Fragiadakis et al., 2025), and is expected to drive a transformation in professional roles rather than straightforward job displacement (Huang and Rust, 2018; Webb et al., 2020). We think that, as in other fields, AI-driven systems could affect the plant breeding sector in the coming years. Rather than job displacement, this transformation is more likely to redefine job tasks, shifting from manually performing analyses and lab operations, and collecting data to supervising, interpreting, and directing the outputs of Agentic AI systems, thereby potentially improving efficiency and decision-making (Chhibber et al., 2025).

## Challenges and limitations

The deployment of AI Agents and Agentic AI in plant breeding is not without significant challenges, spanning technical, ethical, and institutional dimensions (Acharya et al., 2025; Deng et al., 2025; Bandi et al., 2025; Sapkota et al., 2026).

### Data interoperability and quality standards

Breeding data are often distributed across field books, genotyping platforms, phenotyping databases, weather files, pedigree systems, laboratory information systems, spreadsheets, and institutional databases. These data sources frequently use different identifiers, formats, ontologies, and quality-control procedures. An Agentic AI system can only coordinate analyses reliably if it can link entries, plots, samples, environments, genotypes, images, and omics records without ambiguity. Therefore, data standards, persistent identifiers, breeding ontologies, metadata completeness, and database interoperability are prerequisites for safe deployment (Wilkinson et al., 2016). Without them, an agent may integrate the wrong data, producing a technically polished but biologically invalid recommendation. This directly affects the reliability of any agentic system, as inconsistent or poorly standardized inputs will propagate errors through the pipeline, undermining the validity of outputs at every downstream decision point.

Plant breeding pipelines are vulnerable to errors in seed handling, field layout, sample collection, DNA extraction, genotyping, phenotyping, image processing, and data entry. A small mislabeling error can propagate through genomic prediction and reduce selection accuracy. Data-quality agents should be mandatory components of any breeding-oriented Agentic AI system. Pedigree consistency, marker quality, plot identity, field-book integrity, image-label correspondence, sample swaps, missingness, outliers, and unexpected relationships should be checked consistently prior to downstream analyses.

#### Computational inequity

While many of these challenges are shared across sectors, several carry specific implications for breeding programs that deserve careful consideration. From a technical standpoint, the computational demands of AI Agents and Agentic AI systems represent a substantial barrier, particularly for small public breeding programs and smaller private companies operating without access to high-performance computing infrastructure (HPC). This limits the generalizability of any proposed implementation: solutions developed and validated in well-resourced institutions may not be practically deployable in public programs operating under tight computational and financial constraints. This creates an equity gap: well-resourced institutions may rapidly adopt agentic AI with the expectation to accelerate genetic gain, while resource-limited programs, often those working on orphan crops or serving smallholder farmers in developing regions, risk being left behind. Open-source tools, shared data standards, lightweight implementations, cloud-access options, training programs, and cross-institutional collaborations will be important for equitable adoption.

#### Emergent behavior and hallucination

Another technical concern, more characteristic of multi-agent and agentic architectures, is the emergence of unpredictable behaviors arising from interactions among autonomous agents (Trusilo, 2023). These phenomena directly compromise the reliability and validity of AI-systems outputs in breeding contexts. In a breeding context, for example, two agents independently optimizing for yield and disease resistance could converge on a crossing strategy that inadvertently narrows genetic diversity, with neither agent accounting for long-term genetic gain. This connects to a broader, arguably more fundamental, limitation of current agentic systems: their tendency to optimize for short-term objectives at the expense of long-term strategies (Paduraru et al., 2025). LLM-based agents can generate outputs that sound plausible but are incorrect. In plant breeding, hallucinations may manifest as fabricated model results, incorrect interpretations of trial analyses, invented parameter values, misdescriptions of experimental designs, or unsupported recommendations for line advancement or crossing (Ji et al., 2023).

#### Accountability, privacy, and safety

Beyond the technical challenges and limitations, AI Agents and Agentic AI have also shown several ethical challenges. Strong concerns have been raised about data privacy and safety (Sawant, 2025). Accountability is another critical issue, especially for agentic systems operating with high autonomy. When a breeding program fails to meet its target product profile, it may be genuinely unclear whether the failure originated in the AI system, the data it was trained on, or the strategic decisions of the breeders who deployed it. Existing frameworks for assigning responsibility in human-led programs are not readily transferable to contexts where autonomous agents participate in consequential decisions. Closely related is the risk of systematic bias: agentic systems trained on historical selection data may replicate past decisions, including those that reflect outdated priorities or the implicit preferences of previous breeders, rather than optimizing objectively toward current breeding goals. Finally, the opacity of many agentic systems poses a significant institutional barrier.

#### Plant breeding-related limitations

Throughout this perspective, we have drawn on examples from healthcare, logistics, finance, and molecular biology to hypothesize potential applications of AI Agents and Agentic AI in plant breeding. While these analogies are instructive, it is equally important to recognize limitations that are specific to the plant breeding context and that may constrain the transferability of evidence from other domains. A first bottleneck is the current lack of AI and engineering expertise within the plant breeding community. Breeding programs are built around expertise in plant science, genetics, pathology, agronomy, and biostatistics, disciplines that do not traditionally encompass the design and deployment of agentic AI systems. However, this challenge is not insurmountable: strategic collaborations between breeding centers and AI developers, or service agreements with major AI providers, could bridge this gap without requiring breeding programs to develop in-house AI engineering capacity. A second limitation concerns the lack of harmonization across breeding programs and species. Breeding objectives, data structures, and pipeline architectures vary substantially across crops, institutions, and target environments. Unlike domains such as finance or healthcare, where data formats and workflows are more standardized, plant breeding lacks universal data standards. Agentic AI systems will therefore likely need to be tailored to specific crops or clearly scoped objectives, limiting the generalizability of any single implementation. A third and more fundamental constraint is that plant breeding remains inherently field-dependent. Technicians and operators must physically sow, phenotype, and harvest; activities that inevitably introduce human error into the data pipeline. Agentic AI systems will need to be designed with explicit tolerance for such errors, accounting for mislabeling, recording mistakes, and protocol deviations as part of their decision-making logic, rather than assuming clean and consistent data inputs. As discussed in the Operational Optimization section, increased mechanization and robotics may progressively reduce but not eliminate this source of variability. Finally, and perhaps most importantly, resistance to change may represent a significant sociological barrier to adoption. Two decades ago, GS was met with widespread skepticism among breeders worldwide, many of whom preferred field testing over marker-based prediction. Today, GS is widely recognized as a cornerstone of modern breeding strategy. We believe a similar trajectory is plausible for AI Agents and Agentic AI, initial resistance followed by gradual adoption as proof-of-concept implementations demonstrate tangible value in real breeding programs.

## Toward the validation of agentic AI in plant breeding

Because the framework proposed here is conceptual, systematic benchmarking against existing breeding workflows and analytical tools will be required before its practical implementation. Evaluation should extend beyond the predictive performance of individual models and assess the performance of the complete agentic workflow. An initial validation step could rely on *in silico* breeding simulations, where identical breeding objectives and scenarios are evaluated using two strategies: a conventional human-led workflow and a human-supervised Agentic AI framework coordinating multiple specialized AI Agents. Performance could be quantified using task-specific metrics, including predictability, error detection, agreement with expert decisions, maintenance of genetic diversity, computational cost, time requirements, and, most importantly, realized genetic gain. Following successful *in silico* evaluation, pilot studies using empirical breeding data would represent a critical step toward practical implementation. Such studies should be conducted within clearly defined breeding objectives and initially restricted to small-scale breeding pipelines in selected major crops. Human-supervised Agentic AI systems could be evaluated across one or more breeding cycles using rigorous benchmarks. These could include the number of parents selected, number and diversity of crosses generated, predictability, error rates, number of lines advanced, field performance across target traits, maintenance of genetic diversity, and ultimately realized genetic gain. Only after these quantitative evaluations demonstrate consistent advantages, or at least comparable breeding outcomes accompanied by improvements in efficiency, reproducibility, or decision support, should broader implementation of Agentic AI systems across breeding programs be considered.

## Conclusion

AI Agents and Agentic AI should not be viewed as another prediction algorithm for plant breeding. Their potential contribution is broader: they may lead to a supervised orchestration layer that connects field design, data quality, trial analysis, enviromics, genomic prediction, phenomics, multi-omics, parent selection, crossing design, and breeder-decision support. In our view, modern breeding programs may lose efficiency not only due to poor prediction models, but also through disconnected data systems, inconsistent analyses, poor traceability, delayed decisions, and incomplete integration of field, genomic, environmental, and historical information.

The future of these systems in plant breeding should be built around human expertise. Breeders remain the real orchestrators, supported by quantitative geneticists, biometricians, pathologists, physiologists, soil scientists, crop modelers, environmental scientists, and field teams. These AI systems may support experts’ skills, potentially boosting efficiency and reducing errors. In this sense, we hypothesize that the most promising future is not one in which AI replaces breeding expertise, but one in which these AI systems help expert teams coordinate the complexity of modern breeding more effectively.

Achieving this potential will require more than technical development, it will demand a shift in conventional breeding mindset. Just as sowing systems and experimental designs were once adapted to accommodate mechanical harvesters and trait phenotyping, the way breeding data is collected, labeled, structured, and stored may need to evolve to meet the requirements of agentic AI systems.

Indeed, realizing this potential will require coordinated investment in data standardization, interoperability across breeding platforms, interpretable agent outputs, and governance frameworks tailored to the institutional context of both public and private breeding programs. The breeding community, alongside major AI tech companies, has an opportunity to shape how these systems are designed and deployed. We believe that getting that right, with transparency and breeder oversight at the center, could be a meaningful contribution to the long-term sustainability of global food systems.

Ultimately, the framework proposed in this perspective represents a conceptual roadmap rather than an experimentally validated system. Empirical evaluation of AI Agent and Agentic AI implementations in real breeding programs will be essential to determine their true impact on genetic gain, operational efficiency, and breeding decision quality.

## Data Availability

The original contributions presented in the study are included in the article/supplementary material, further inquiries can be directed to the corresponding authors.
